# Elevated filling pressures are associated with poor long-term graft survival after pediatric heart transplantation

**DOI:** 10.3389/ti.2026.16339

**Published:** 2026-06-15

**Authors:** Ezgi Yavasca, Lisa-Maria Rosenthal, Regina Stegherr, Levin Wiebelt, Isabell Just-Lauer, Peter Kramer, Friederike Danne, Felix Schoenrath, Frank Konietschke, Mustafa Yigitbasi, Felix Berger, Katharina R. L. Schmitt, Oliver Miera, Fatima I. Lunze

**Affiliations:** 1 Deutsches Herzzentrum der Charite Klinik für Angeborene Herzfehler - Kinderkardiologie, Berlin, Germany; 2 Charite - Universitatsmedizin Berlin, Berlin, Germany; 3 Deutsches Zentrum fur Herz-Kreislauf-Forschung eV, Berlin, Germany; 4 Institute of Biometry and Clinical Epidemiology, Charité - Universitätsmedizin Berlin, Berlin, Germany; 5 Deutsches Herzzentrum der Charite Klinik fur Herz- Thorax- und Gefasschirurgie, Berlin, Germany

**Keywords:** cardiovascular outcomes, coronary allograft vasculopathy, filling pressures, heart transplantation, pediatrics, pulmonary capillary wedge pressure, right atrial pressure

## Abstract

Long-term survival has improved in the current era of pediatric heart transplantation (HT). The impact of elevated filling pressures [EFP; defined as pulmonary capillary wedge pressure (PCWP) > 15 mmHg and/or right atrial pressure (RAP) > 12 mmHg in the absence of biopsy-confirmed rejection] on long-term outcomes beyond 10 years remains poorly characterized. We assessed whether EFP during the early years after HT are associated with poor graft survival and cardiovascular adverse events (AE). We retrospectively analyzed 114 pediatric HT grafts (1986–2020) with available PCWP and/or RAP measurements 7 months to 5 years post-transplant (grouping period), representing a landmark cohort of 5-year survivors. Associations of EFP with graft survival and AE were evaluated. Fourteen grafts (12%) had EFP during the grouping period. Grafts with EFP had significantly worse long-term survival (44% vs. 85% at 10 years; log-rank p < 0.001), and higher risk of graft loss (overall HR 6.04, 95% CI [2.01–16.85]). The incidence of AE was numerically higher in grafts with EFP (26.6 [15.2–43.2] vs. 11.9 [9.4–14.9] per 100 person-years), but should be interpreted as exploratory. EFP within the early years post-transplant are associated with poor graft survival and may indicate cardiovascular complications.

## Graphical Abstract

**Figure:**
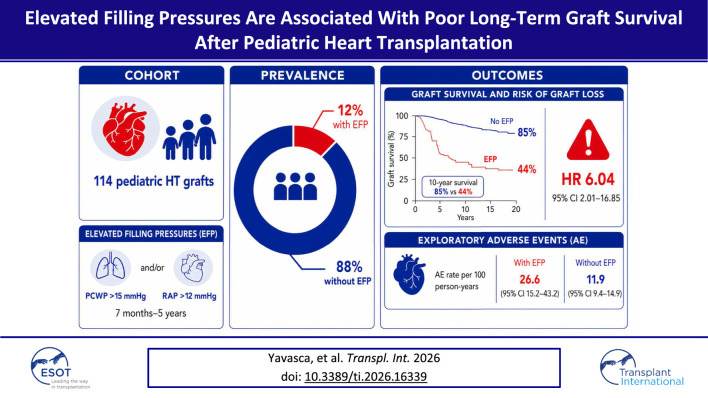

## Introduction

Heart transplantation (HT) is an established and effective therapy for children with end-stage heart failure. Approximately 650 pediatric transplantations are performed annually, with more than 14.000 recipients worldwide [1]. Advances in surgical techniques and immunosuppression have resulted in excellent short-term outcomes, with first-year survival rates approaching 90%, and long-term survival beyond 10 years became increasingly common in the current pediatric HT era, with median post-transplant survival of approximately 15 years across all pediatric age groups [2]. Nevertheless, overall graft longevity remains limited, and late graft failure continues to represent a major clinical challenge [2, 3].

Coronary allograft vasculopathy (CAV) is a well-established contributor to late graft failure and morbidity [2]. It is characterized by diffuse intimal proliferation and maladaptive vascular remodeling affecting both the epicardial coronary arteries and the microvasculature, ultimately leading to graft dysfunction and failure [4–13]. However, CAV alone does not fully explain the heterogeneity of long-term outcomes among recipients who survive in the late post-transplant period. In particular, restrictive physiology and microvascular dysfunction have been proposed as additional contributing factors and have been associated with adverse outcomes [8, 12–14].

According to the current ISHLT guidelines, cardiac catheterization with coronary angiography remains the gold standard for surveillance of epicardial CAV, yet reliable clinical tools to assess microvascular function are lacking [15, 16]. While elevated filling pressures (EFP), reflected by increased pulmonary capillary wedge pressure (PCWP) and right atrial pressure (RAP), may represent a hemodynamic correlate of underlying graft pathology, their relationship with microvascular remains incompletely understood, primarily associative and inferential [15]. Current guidelines address markedly elevated pressures (PCWP >25 mmHg and/or RAP >12 mmHg), while the 2023 update incorporates pediatric data from Kindel et al. suggesting that EFP defined by PCWP >15 mmHg and/or RAP >12 mmHg may already be clinically relevant [15–17]. Despite these observations, the prognostic significance of EFP for long-term outcomes after HT and their relationship with survival beyond epicardial CAV, remain poorly explored in pediatric recipients. Therefore, we investigated whether EFP during the early years after pediatric HT are associated with poor long-term survival and cardiovascular adverse events (AE).

## Materials and methods

### Study population and design

We screened all pediatric heart recipients who underwent primary HT and/or re-transplantation (re-HT) before 18 years of age between 1986 and 2020 at our institution and were followed thereafter. All patients underwent orthotopic HT using either biatrial or bicaval technique with ABO-compatible donor-recipient matching. Inclusion required at least one cardiac catheterization with invasive measurement of PCWP and/or RAP within the grouping period, defined as 7 months to 5 years after HT. Grafts without right-sided hemodynamic data during this period were excluded. Patients were excluded if they underwent multi-organ transplantation or if grafts failed to reach the 5-year landmark due to mortality or follow-up shorter than 5 years (Supplementary Figure S1). This study was approved by the Institutional ethics committee of Charité - Universitätsmedizin Berlin (EA2/055/23).

All patients received induction therapy with methylprednisolone combined with either a polyclonal lymphocyte or thymocytic antibody or basiliximab. Presensitized recipients additionally underwent plasmapheresis. Until 2000, maintenance immunosuppression consisted of a triple regimen with corticosteroids, cyclosporin A and azathioprine. From 2000 onward, recipients were treated with mycophenolate mofetil in combination with a calcineurin inhibitor, either cyclosporin A or tacrolimus, during the first year post-transplant. After the first year, immunosuppression typically consisted of a calcineurin inhibitor combined with either mycophenolate mofetil or everolimus.

This is a single-center retrospective cohort study. Data was analyzed between September 2024 and July 2025. The primary predictor was EFP defined as PCWP >15 mmHg and/or RAP >12 mmHg [17], excluding measurements during acute rejection episodes confirmed by concurrent endomyocardial biopsy (EMB) (≥2R/3A acute cellular rejection [ACR] and/or antibody-mediated rejection [AMR]) according to ISHLT – 1990 and 2004 guidelines [18–20]. EFP status was assigned if present in at least one catheterization within the grouping period and may therefore have been influenced by variability in catheterization frequency and timing. Thresholds were applied to values from each examination without further adjudication. There was no averaging across catheterizations. Multiple measurements within a single catheterization were averaged. Grafts were classified into EFP and no-EFP groups accordingly and compared regarding survival and AE. AE were defined as a composite endpoint comprising moderate-severe epicardial CAV_2-3_, myocardial infarction (MI) and/or coronary revascularization, arrhythmias, and non-rejection heart failure hospitalization. Graft loss was defined as death or re-HT due to graft dysfunction.

The 7-month lower bound of the grouping period was chosen to avoid perioperative confounding factors such as graft adaptation, cold ischemic time, and early rejection [21, 22]. Landmark approach was chosen because a uniform baseline assessment was not available and catheterizations varied by timing and content over the study period. In some examinations, only left heart catheterization (LHC) was performed, without right-sided hemodynamics (PCWP/RAP). Accordingly, grafts without any available PCWP/RAP within the grouping period were excluded. The 5-year upper bound was chosen because surveillance catheterizations were most consistently performed during this period, whereas beyond 5 years catheterizations were less frequent and mainly clinically indicated and a shorter time window yielded too few EFP events for stable estimates. This broad window enabled classification despite variability in right heart catheterization (RHC) timing. However, using a 5-year landmark conditioned analyses on graft survival, excluding early graft failures and defining a selected cohort with available hemodynamics.

### Institutional graft surveillance

Invasive catheterization-based graft surveillance was performed as part of the institutional surveillance protocol including LHC with coronary angiography and RHC with EMB and measurement of PCWP/RAP beginning one-year post-transplant. Following the first year, LHC with coronary angiography and RHC with EMB was scheduled every 1–2 years during the first 5 years and at least every 2 years thereafter. Catheterization was also performed whenever clinically indicated. Procedures were almost exclusively performed under analgosedation with spontaneous breathing.

Coronary arteries were visualized by either aortography in children <10 kg or selective coronary angiography in children >10 kg. The presence of CAV was graded according to the 2010 ISHLT classification [15].

Until 2004, moderate and severe ACR were classified as grade 3A and ≥ 3B according to the ISHLT 1990 guideline [18, 20] from 2004 onward, they were classified as grade 2R and 3R according to the ISHLT 2004 guideline, respectively [19, 20]. AMR was considered when at least three of the following four ISHLT criteria were evident: histological or immunological evidence of antibody-mediated rejection, new-onset graft dysfunction, and donor-specific antibodies [23, 24]. After transition to adult HT care, the intervals between catheterizations were individually adjusted depending on individual risk profile, clinical status, graft performance, and time after HT.

Echocardiographic examinations were performed as part of non-invasive graft surveillance on the day of catheterization and during routine outpatient visits according to current echocardiographic guidelines [25, 26]. Left ventricular systolic function was assessed by calculating left ventricular ejection fraction (LVEF) using the biplane Simpson method, and two-dimensional–guided M-mode echocardiography when Simpson measurements were not available. LVEF was classified as preserved (≥50%), mildly reduced (40%–49%), or reduced (<40%) [25–28]. Advanced echocardiographic parameters (e.g., strain imaging) could not be included in the analysis, given their limited availability due to the long study period and evolving echocardiographic practice.

### Statistical analysis

For practicability, grafts were treated as independent observations. Categorical variables were expressed as absolute and relative frequencies, and continuous variables as medians with interquartile ranges (IQRs). Demographic parameters were calculated over the whole study period, including pre-grouping (0–7 months), grouping (7 months–5 years), analysis (5–13.8 years), and post-analysis period (13.8–35 years). The post-analysis period begins once all grafts in one group have experienced graft loss or were censored. As no meaningful comparison is possible once one group is depleted, graft survival and AE analyses were restricted to the analysis period.

Exposure status (EFP vs. no-EFP) was determined based on whether filling pressures were elevated at least once within the grouping period. Survival analyses were conducted using a landmark approach, beginning 5 years post-transplant, so that both groups entered the analysis period uniformly. Some grafts initially classified as no-EFP subsequently developed EFP beyond 5 years post-transplant. Sensitivity analysis excluded these late converters, redefining the comparator as never-EFP group. Survival of grafts with EFP was then compared with that of grafts that never developed EFP. Grafts were administratively censored at 13.8 years, corresponding to the maximum graft survival time in the EFP group. Survival functions were estimated using the Kaplan–Meier method and compared using log-rank test. Raw graft lifespan up to 31 years were also reported. A Cox proportional hazard model quantified effect size (hazard ratio) adjusting for transplantation era (≤1999 vs. ≥ 2000) and recipient age at HT. Confidence intervals of the main results were calculated by an accelerated bootstrap to adjust for overfitting. Multiple sensitivity analyses were performed to assess robustness to the proportional hazards assumption which was violated by main factor (EFP group) and covariates (transplantation era and recipient age). Two piecewise Cox models fitted to estimate hazard ratios for the 5–9 and 9–14 years post-transplant periods. Additionally, risk ratios were calculated from cumulative hazards for the timepoints 7 and 11 years. Recipient age at HT was dichotomized at three different cut-offs (2 years, 10 years, and 15 years).

AE during the analysis period were analyzed using incidence rates. Since observation time varied by graft, absolute event counts are misleading; therefore, event rates were standardized per 100 patient-years. Patient-years for a graft was calculated as time from the 5-year landmark until the last observation or censoring time (13.8 years), whichever came first. This approach incorporated recurrent events. Confidence intervals and explorative p-values were calculated based on the Poisson distribution. To assess robustness to overdispersion, rate ratios were additionally calculated using the negative binomial distribution. Moreover, a responder analysis compared the proportion of grafts with ≥1 AE during the grouping and analysis period. Grafts alive at study end, or lost to follow-up, were censored at the date of last patient contact.

All tests were two-sided and performed with R version 4.4.1, using the packages “survival” (3.6.4), “boot” (1.3.30), and “MASS” (7.3.64).

## Results

### Study population

Of 203 grafts from 193 patients, 43 grafts lacked digitized medical records available for review. Three grafts from patients who underwent re-HT after the age of 18, 24 grafts that failed to reach the 5-year landmark due to mortality or follow-up shorter than 5 years, and 19 grafts without RHC documenting PCWP and/or RAP during the grouping period were excluded (Supplementary Figure S1). A comparison of demographic and clinical characteristics between included and excluded grafts are provided as Supplementary Tables S1, S2. The final analysis cohort therefore included 114 grafts from 112 patients.

**FIGURE 1:**
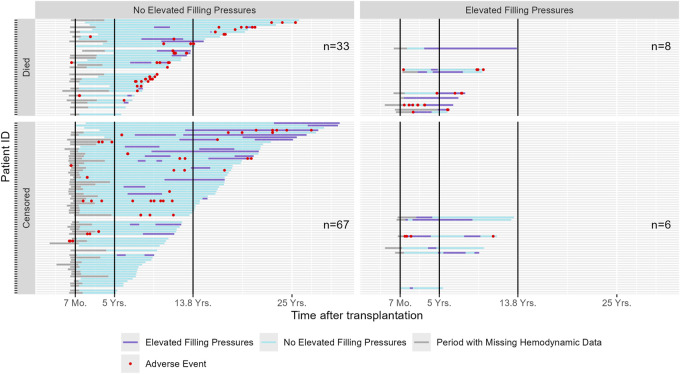
Swimmer plot showing individual graft follow-up trajectories from heart transplantation to graft loss or censoring, along with cardiovascular AE in both groups. Each horizontal bar represents a single graft. Color segments indicate periods with elevated filling pressures (EFP) (purple), no EFP (blue), or periods without available hemodynamic data (gray). The length and timing of these segments vary between grafts due to the retrospective nature of the cohort, reflecting differences in the timing and frequency of surveillance; early gray segments indicate periods prior to the first available hemodynamic assessment. Vertical lines mark key analysis time points: 7 months (start of grouping window), 5 years (landmark for survival analysis), and 13.8 years (end of analysis period). Red dots represent adverse events. Panels separate grafts that died from those censored, and cohorts are stratified by EFP vs. no-EFP status.

12% of the grafts (14/114) had ≥1 EFP within the grouping period (Figure 1). Over the entire follow-up period, EFP were diagnosed in 54% of the grafts (61/114) with a median onset of 6.8 years (IQR 2.0–10.6) post-transplant. Freedom from EFP was 87% at 2 years, 80% at 5 years, 61% at 10 years, and 47% at the end of the observation period.

The demographic characteristics are summarized in Table 1. Median age at HT for the cohort was 8.9 years with median follow-up time of 13.1 years. Compared with no-EFP, EFP recipients were slightly older at HT (median 12.2 vs. 8.8 years) however, age difference was not significant. Donor and recipient age difference was also not significantly different between both groups. Era distribution was similar across groups, with most transplantations performed in the most recent era 2000–2020. The EFP group had more digitally available catheterizations than the no-EFP group (median 9.0 vs. 7.0). The time to first catheterization with available filling pressures did not differ significantly between groups (median 1.1 vs. 1.5 years, p = 0.215). A preserved LVEF (≥50%) was present in 64% of EFP vs. 72% of no-EFP grafts, while a reduced systolic left ventricular function with an LVEF <40% was present in 29% and 12% of grafts, respectively. Overall, 36% of grafts had ≥1 acute rejection (≥2R/3A ACR and/or AMR), occurring at similar rates and comparably distributed in both groups, with most episodes arising after the first catheterization with available filling pressures.

**TABLE 1:** Demographic and clinical characteristics of grafts, stratified by EFP status.

Variables	Total (n = 114)	No-EFP group (n = 100)	EFP group (n = 14)	p-value
Recipients’ age at HT/re-HT, years	8.9 (2.7–14.3)	8.8 (2.9–14.2)	12.2 (2.7–14.5)	0.769
Post-transplant follow up, years	13.1 (8.9–17.7)	13.9 (9.3–18.3)	9.7 (6.7–11.1)	0.002
Donors’ age, years	9 (3–24)	9 (3–20.5)	12 (3–31.2)	0.861
Donor/recipient age difference, years	1.8 (−0.7–8.7)	1.8 (−0.8–7.1)	1.6 (−0.1–15.5)	0.583
Gender Female	51 (45%)	47 (47%)	4 (29%)	0.312
Number of re-HT	3 (3%)	2 (2%)	1 (7%)	0.815
Eras of transplantation: • Early era (1986–1999) • Late era (2000–2020)	34 (30%) 80 (70%)	31 (31%) 69 (69%)	3 (21%) 11 (79%)	0.674
*C*ardiac diagnosis prior HT: • Cardiomyopathy • Congenital heart disease • Others • Re-HT	94 (83%) 14 (12%) 3 (3%) 3 (3%)	82 (82%) 14 (14%) 2 (2%) 2 (2%)	12 (86%) - 1 (7%) 1 (7%)	0.217
Time from HT to first catheterization with available FP, years	1.4 (1.0–2.9)	1.5 (1.0–2.9)	1.1 (1.0–1.9)	0.215
Number of catheterizations per graft	7 (5–9)	7 (5–9)	9 (7–15)	0.006
LVEF echocardiographic evaluation* ⁃ Grafts with preserved pLVEF ≥50% ⁃ Grafts with mildly reduced mrLVEF = 40–49% ⁃ Grafts with reduced rLVEF <40%	81 (71%) 17 (15%) 16 (14%)	72 (72%) 16 (16%) 12 (12%)	9 (64%) 1 (7%) 4 (29%)	0.207
Acute rejection episodes ≥ 1** ⁃ From HT to the first catheterization with FP ⁃ Thereafter to the last follow-up or death/re-HT	41 (36%) 11 (10%) 30 (26%)	36 (36%) 10 (10%) 26 (26%)	5 (36%) 1 (7%) 4 (29%)	1.000
PTLD	11 (10%)	11 (11%)	-	0.411

EFP, Elevated filling pressures; FP, Filling pressures HT, Heart transplantation; LVEF, Left ventricular ejection fraction. PTLD, Post-transplant lymphoproliferative disorder; re-HT, Re-transplantation.

* For LVEF, values indicate the number of grafts categorized according to their lowest recorded EF during follow-up: pLVEF: preserved, always ≥50%, mrLVEF: mildly reduced, at least once <50% but never <40%, rLVEF: reduced, at least once <40% over the whole study period.

** For acute rejection, values indicate the number of grafts that experienced ≥1 biopsy-proven episodes (≥2R/3A ACR and/or AMR).

Baseline characteristics were comparable between eras (Table 2). Follow-up time was longer in the earlier era (median 14.7 vs. 12.4 years, p = 0.007). Time to first catheterization with available filling pressures was not significantly different.

**TABLE 2:** Baseline characteristics and catheterization patterns by transplantation era.

Variables	1986–1999 (n = 34)	2000–2020 (n = 80)	Total (n = 114)	p-value
Recipients’ age at HT/re-HT, years	10.5 (5.8–14.5)	8.7 (2.4–14)	8.9 (2.7–14.3)	0.227
Post-transplant follow up, years	14.7 (10.2–25.4)	12.4 (8.1–16.9)	13.1 (8.9–17.7)	0.007
Donors’ age, years	9 (3.8–22.8)	9 (3–23.5)	9 (3–24)	0.588
GenderFemale	15 (44%)	36 (45%)	51 (45%)	1.000
Number of re-HT	-	3 (4%)	3 (3%)	0.614
Number of catheterizations per graft	8 (4–9)	7 (5–9)	7 (5–9)	0.784
Time from HT to first catheterization with available FP, years	2.5 (1.5–3.8)	1.2 (1.0–1.9)	1.4 (1.0–2.9)	0.215
Time from HT to first catheterization, years	1.6 (0.4–3.1)	1.0 (0.2–1.3)	1.0 (0.3–1.7)	0.031
Proportion of catheterizations with available FP	0.73 (0.56–1.0)	1.0 (0.83–1.0)	1.0 (0.8–1.0)	<0.001
Number of grafts with EFP	3 (9%)	11 (14%)	14 (12%)	0.674

EFP, Elevated filling pressures; FP, Filling pressures; HT, Heart transplantation; re-HT, Re-transplantation.

The proportion of catheterizations including PCWP and/or RAP measurements was lower in the earlier era (median 0.73 vs. 1.00), indicating more complete hemodynamic assessment in recent years.

Overall, graft loss occurred in 36% of the grafts (41/114), affecting 35% of the patients (39/112). Among 41 graft losses, causes were cardiac in 51% (21/41), non-cardiac in 7% (3/41), and unknown in 42% (17/41), respectively (Table 3).

**TABLE 3:** Distribution of deaths by cause and time interval after pediatric heart transplantation.

Characteristics	Total (n = 114)	No-EFP group (n = 100)	EFP group (n = 14)
Number of deaths during entire study period	41 (36%)	33 (33%)	8 (57%)
Cardiac deaths: • CAV and/or acute rejection • Heart failure Noncardiac deaths: • PTLD • Infection/Sepsis Unknown	13 (11%) 8 (7%) 1 (1%) 2 (2%) 17 (15%)	12 (12%) 7 (7%) 1 (1%) 1 (1%) 12 (12%)	1 (7%) 1 (7%) - 1 (7%) 5 (36%)
Number of deaths within analysis period (5–13.8 years)	29 (25%)	21 (21%)	8 (57%)
Cardiac deaths: • CAV and/or acute rejection • Heart failure Noncardiac deaths: • PTLD • Infection/Sepsis Unknown	11 (10%) 6 (5%) 1 (1%) 2 (2%) 9 (8%)	10 (10%) 5 (5%) 1 (1%) 1 (1%) 4 (4%)	1 (7%) 1 (7%) - 1 (7%) 5 (36%)
Number of deaths within post analysis period (after 13.8 years)	12 (11%)	12 (12%)	-
Cardiac deaths: • CAV and/or acute rejection • Heart failure Noncardiac deaths: • PTLD • Infection/Sepsis Unknown	2 (2%) 2 (2%) - - 8 (7%)	2 (2%) 2 (2%) - - 8 (8%)	- - - - -

CAV, Coronary allograft vasculopathy EFP, Elevated filling pressures; PTLD, Post transplant lymphoproliferative disorder.

### Survival analysis

**FIGURE 2:**
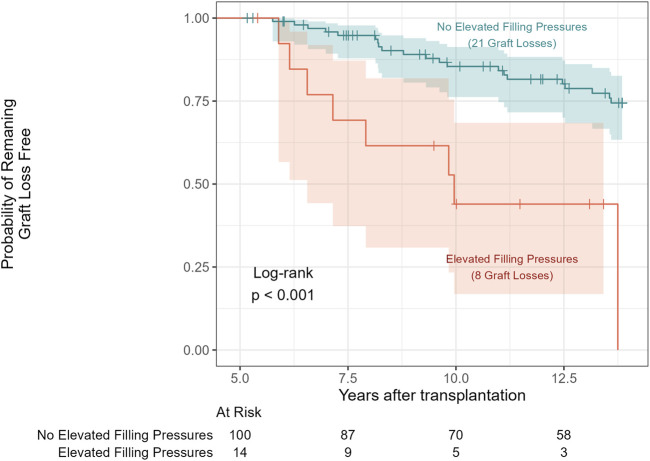
Graft survival comparing grafts with EFP to those without EFP during the analysis period. Kaplan–Meier curves estimate graft loss-free survival from the 5-year landmark to 13.8 years after transplantation, comparing grafts with elevated filling pressures (EFP) to those without EFP during the analysis period. Shaded areas represent 95% confidence intervals. Tick marks denote censoring. The log-rank test (p < 0.001) indicates significantly worse graft survival in the EFP group. Numbers at risk at selected time points are presented below the curves.

The comparison of graft survival over time is illustrated in Figure 2. Survival probability for grafts in the EFP group considerably decreased from year 6 onward. At 10 years post-transplant, graft survival was significantly lower in the EFP group (44%, 95% CI 16.8–68.4) compared with no-EFP group (85%, 95% CI 76.2–91.3, log-rank p < 0.001).

**FIGURE 3:**
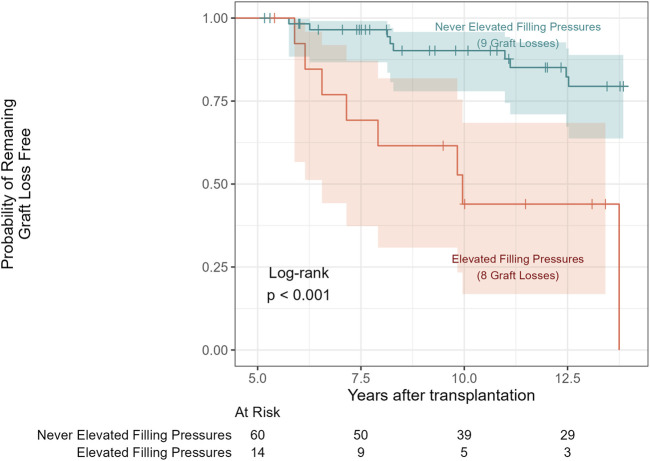
Sensitivity analysis of graft survival using a never-EFP comparator group. Kaplan–Meier curves estimate graft survival excluding grafts from the no-EFP group that developed elevated filling pressures (EFP) after the 5-year landmark (reclassified as never-EFP group). Shaded areas represent 95% confidence intervals. Tick marks denote censoring. Kaplan–Meier curves demonstrate persistent survival differences, with significantly worse graft survival in grafts EFP (log-rank p < 0.001). Numbers at risk at selected time points are presented below the curves.

In a sensitivity analysis excluding grafts from the no-EFP group that developed EFP beyond 5 years post-transplant (redefined as never-EFP group), grafts with EFP showed particularly worse survival compared with those that never developed EFP (log-rank p < 0.001) (Figure 3). A comparison of grafts that never developed EFP and those with EFP, including demographics, clinical characteristics, and cardiovascular AE, is provided in Supplementary Tables S3, S4.

In Cox regression analyses, EFP were associated with a higher hazard of graft loss (overall HR = 6.04, 95% bootstrap-CI 2.01–16.85), representing a summary estimate across follow-up. This association remained consistent across stratified and piecewise Cox models, with higher effect estimates observed during later follow-up periods (9–14 years post-transplant HR = 10.15, 95% CI 2.19–46.93) (Table 4). Cumulative risk estimates supported this association, demonstrating a 5.5-fold higher risk of graft failure at 7 years (95% CI 1.4–16.6) and a 3.5-fold higher risk at 11 years (95% CI 1.4–6.9) in grafts with EFP.

**TABLE 4:** Cox regression estimates with 95% confidence intervals.

Model	HR	CI
EFP-HR with Bootstrap-CI (main analysis) Sensitivity analyses EFP-HR with Wald-CI EFP-HR stratified by era, age ≥2 EFP-HR stratified by era, age ≥10 EFP-HR stratified by era, age ≥15 EFP-HR piecewise, year 5–9 EFP-HR piecewise, year 9–14	6.04 6.04 6.03 6.08 6.40 6.04 10.15	[2.01, 16.85] [2.51, 14.54] [2.48, 14.67] [2.51, 14.73] [2.62, 15.63] [2.51, 14.54] [2.19, 46.93]

CI, Confidence intervals; EFP, Elevated filling pressures HR, Hazard ratio.

### Cardiovascular AE

The timing of EFP and AE occurrence is illustrated in Figure 1. During the grouping period, ≥1 AE occurred in 5/14 (36%) of EFP vs. 7/100 (7%) of no-EFP grafts (p = 0.005). Several component rates were higher in the EFP group: CAV_2-3_ in 4/14 (29%) vs. 1/100 (1%), MI/revascularization in 4/14 (29%) vs. 2/100 (2%) (Table 5).

**TABLE 5:** Cardiovascular adverse events within the grouping and analysis periods after pediatric heart transplantation, stratified by EFP status.

Variables:	Total (n = 114)	No-EFP group (n = 100)	EFP group (n = 14)	p-value**
Within grouping period (7 months - 5 years post-transplant)*				
Cardiovascular AE Composite Endpoint ≥ 1: ⁃ Moderate - severe epicardial CAV_2-3_ ⁃ Cardiac arrhythmia ⁃ Non-rejection heart failure hospitalization ⁃ MI and/or coronary revascularization	12 (11%) 5 (4%) 5 (4%) 1 (1%) 6 (5%)	7 (7%) 1 (1%) 5 (5%) 0 (0%) 2 (2%)	5 (36%) 4 (29%) - 1 (7%) 4 (29%)	0.005 <0.001 0.874 0.248 <0.001
Within analysis period (5–13,8 years post-transplant)*				
Cardiovascular AE Composite Endpoint ≥ 1: ⁃ Moderate - severe epicardial CAV_2-3_ ⁃ Cardiac arrhythmia ⁃ Non-rejection heart failure hospitalization ⁃ MI and/or coronary revascularization	24 (21%) 17 (15%) 7 (6%) 6 (5%) 12 (11%)	20 (20%) 14 (14%) 6 (6%) 5 (5%) 9 (9%)	4 (29%) 3 (21%) 1 (7%) 1 (7%) 3 (21%)	0.699 0.741 1.000 1.000 0.340

AE, Adverse events; CAV, Coronary allograft vasculopathy EFP, Elevated filling pressures; MI, Myocardial infarction.

* Values represent the number of grafts (n) with at least one event, with percentages (%) referring to the respective group. Each graft is counted once per event category. Grafts may have experienced more than one event type, and multiple events within the same graft were possible.

** With event counts lower than 5, p-value violates assumption of chi-square test.

In the analysis period, 4/14 (29%) of the EFP grafts and 20/100 (20%) of the no-EFP grafts experienced ≥1 AE. EFP grafts showed numerically higher proportions of CAV_2-3_ (3/14 [21%] vs. 14/100 [14%]) and MI/revascularization (3/14 [21%] vs. 9/100 [9%]), although this difference was not statistically significant. Arrhythmia (1/14 [7%] vs. 6/100 [6%]) and non-rejection heart failure hospitalization (1/14 [7%] vs. 6/100 [5%])rates were similar.

**FIGURE 4:**
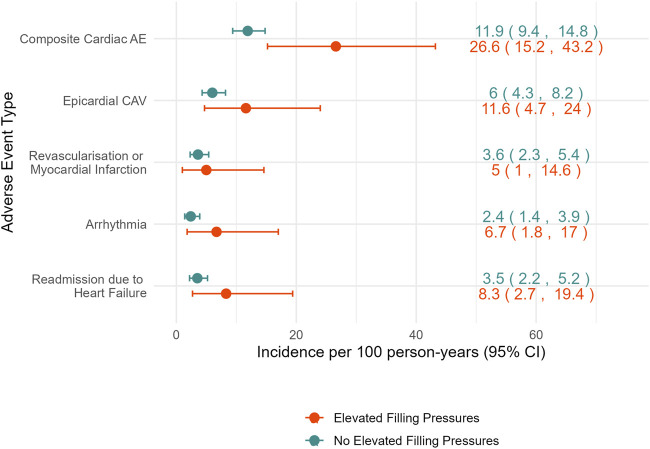
Comparison of incidence rates (per 100 person-years, 95% CI) of composite cardiovascular AE during the analysis period (5–13.8 years post-transplant) between grafts with EFP and those without. Point estimates and confidence intervals are shown for the composite cardiovascular adverse events (AE) endpoint and individual components: moderate - severe epicardial coronary allograft vasculopathy (CAV_2-3_), myocardial infarction (MI) and/or revascularization, arrhythmia, and non-rejection heart failure hospitalization. Incidence rates account for varying follow-up time and include recurrent events.

The overall AE incidence was 26.6 events per 100 patient-years (95% CI 15.2–43.2) in the EFP group vs. 11.9 per 100 patient-years (95% CI 9.4–14.9) in the no-EFP group (Figure 4). Component-specific incidence rates with overlapping confidence intervals are presented in Figure 4. Sensitivity analyses using negative binomial models showed that these results are not robust to the overdispersion observed in the data and should therefore be considered exploratory (Supplementary Table S5). In these analyses, attenuation was most pronounced for CAV_2-3_, MI/revascularization and non-rejection heart failure hospitalization, whereas findings for composite AE and arrhythmia were more consistent.

## Discussion

In this single-center cohort of pediatric HT recipients, the presence of EFP within the first years post-transplant was strongly associated with poor long-term graft survival. The association remained significant after adjustment for transplantation era and recipient age at HT and was robust across sensitivity analyses. However, given the violation of the proportional hazards assumption, the hazard ratio reflects a time-varying summary estimate. Despite potential residual confounding, these findings suggest that EFP is associated with increased long-term graft loss.

Multiple adult HT studies have demonstrated that adverse hemodynamic profiles characterized by elevated RAP and PCWP and reduced cardiac index are associated with mortality, graft failure, and CAV beyond angiographic findings [29–34]. In a recent cohort, patients with restrictive hemodynamics and heart-failure symptoms had worse survival than CAV_3_ alone, underscoring the prognostic value of hemodynamics [34].

Our results are consistent with these previous findings, despite differences in applied thresholds and pediatric physiology. Pediatric data on this topic remain limited [17, 35]. Early work by Aiyagari et al. showed an association between higher filling pressures and graft loss, as well as with epicardial CAV [35]. In a large multicenter cohort of over 3,100 pediatric HT recipients, Kindel et al. proposed pediatric-specific thresholds and showed that children with CAV_1_ and a single hemodynamic alteration had significantly worse survival [17].

Our study extends these findings by showing that EFP are associated with poor graft survival with follow-up spanning up to three decades and uniquely capturing outcomes from childhood through adulthood. While registry studies provide broader generalizability, they often lack data granularity. The single-center design allowed detailed procedural control, including exclusion of measurements obtained during biopsy-proven rejection, and enabled graft-level analysis within a prespecified exposure window. We acknowledge trade-offs, including a smaller sample size and limited generalizability however our data complement existing registry evidence by clarifying the timing, measurement context, and clinical interpretation of EFP in pediatric HT.

Grafts with EFP showed a numerically higher incidence of AE. However, confidence intervals were wide, particularly in the EFP group due to the small sample size and results were sensitive to overdispersion in sensitivity analyses. Additionally, absolute and component-level differences were not statistically significant. Accordingly, these findings related to AE should be considered exploratory and hypothesis-generating. Despite the paucity of research explicitly correlating EFP to AE, our exploratory findings point in the same direction as adult data linking abnormal hemodynamics, particularly elevated PCWP and RAP during exercise, to adverse outcomes [36].

Our study encompasses a long period (1986–2020), during which substantial changes in immunosuppression, surveillance practices, and data availability may have influenced both EFP detection and outcomes. These changes raise the possibility of era-related ascertainment bias, with EFP potentially more frequently identified in recent eras with more consistent hemodynamic assessment. Differences in surveillance intensity may have further influenced exposure classification. Grafts classified as EFP had more frequent catheterizations, increasing the possibility of detecting EFP, whereas grafts with fewer assessments may have had undetected EFP, introducing potential misclassification. Consistent with these considerations, EFP grafts were more frequently observed in recent eras, whereas many no-EFP grafts were transplanted in earlier eras, in which survival is generally poorer both at our center [37] and in registry reports [2]. Despite this distribution, survival remained consistently superior in the no-EFP group, arguing against era effects as the sole explanation for the observed association. To address this, we adjusted for transplantation era in Cox models. This adjustment, along with sensitivity analyses, did not alter the direction of the association between EFP and graft loss, suggesting that ascertainment differences alone are unlikely to fully explain the findings. As not all relevant confounders were included (graft function, CAV burden, rejection history, donor–recipient characteristics, changes in immunosuppressive regimens and surveillance intensity), residual confounding and ascertainment bias cannot be excluded. The observed associations should therefore be interpreted with appropriate caution as adjusted for selected covariates rather than fully independent.

The study design excluded the early post-transplant period to reduce perioperative confounding and minimize lead-time and immortal time bias and conditioned analyses on survival to the 5-year landmark, thereby excluding early graft failures. Grafts lacking qualifying hemodynamic assessments were also excluded. Accordingly, the cohort represents a selected population of 5-year survivors with available hemodynamic data, limiting generalizability to earlier post-transplant outcomes and the broader pediatric transplant population.

Although EFP may reflect underlying graft pathology including potential microvascular involvement, these interpretations remain speculative, as the mechanisms cannot be directly inferred from hemodynamic measurements and reliable tools to assess microvascular function are lacking. Overall, these findings suggest a potential clinical relevance of early recognition of EFP in pediatric recipients.

Systolic function was primarily assessed by LVEF using the Simpson method or, when unavailable, M-mode which may overestimate systolic function in transplanted hearts due to geometric assumptions. LVEF also has limited sensitivity for early myocardial dysfunction. Advanced parameters such as global longitudinal strain (GLS) and left atrial strain (LAS) may provide more sensitive assessment [38, 39] with LAS showing a superior correlation with PCWP [40–44]. These parameters were not consistently available across the cohort, particularly in earlier eras, and therefore could not be included in the analysis. Their integration into future studies may improve mechanistic understanding and risk stratification.

Our study highlights the importance of structured long-term follow-up in pediatric heart transplant recipients, particularly during transition to adult care. The findings suggest that EFP, when assessed during routine evaluation, may help identify grafts at increased long-term risk. In this context, hemodynamic parameters may complement existing surveillance approaches, although their role in guiding follow-up strategies requires further investigation. Beyond surveillance, identifying the underlying substrates of EFP remains critical, as this may provide insight into graft pathophysiology. Future studies should aim to clarify the mechanisms contributing to EFP to enable more personalized long-term post-transplant management especially tailored to childhood-onset recipients.

### Limitations

Taken together, this study has several important limitations. First, its single-center, retrospective design and the relatively small number of grafts with EFP limit generalizability, particularly for cardiovascular AE, which require confirmation in larger, adequately powered cohorts. Second, exclusion of grafts without retrievable hemodynamic data and conditioning on survival to the 5-year landmark may have introduced selection and ascertainment bias, enriching the cohort for later eras with more consistent surveillance. Practice patterns evolved over the 35-year study period, introducing potential heterogeneity in post-transplant surveillance. The retrospective design relied on routinely collected data, resulting in missing variables, and institutional follow-up may have led to under-capture of external events. A substantial proportion of graft losses were of unknown cause, limiting mechanistic attribution. A small number of patients contributed more than one graft, analyzed as independent observations, which may underestimate within-patient correlation, although the impact is likely minimal. Advanced assessments of microvascular function, including index of microcirculatory resistance, coronary flow reserve, or biopsy-based techniques, were unavailable. Therefore, any association between EFP and microvascular CAV remains inferential. Collectively, these limitations indicate that the findings should be considered hypothesis-generating and warrant validation in larger multicenter, prospective studies.

## Conclusion

The presence of EFP between 7 months and 5 years after pediatric HT is associated with significantly poorer long-term graft survival and potentially increased cardiovascular AE, although findings related to AE remain exploratory. These results suggest that EFP may help identify grafts at increased long-term risk. The clinical implications for surveillance strategies and therapeutic decision-making require further investigation. Future multicenter studies are needed to validate these findings, further elucidate underlying mechanisms, and determine their potential impact on long-term outcomes.

## Data Availability

The raw data supporting the conclusions of this article will be made available by the authors, without undue reservation.
