# Supplementary figures and images for: Elevated filling pressures are associated with poor long-term graft survival after pediatric heart transplantation
Source: Transpl Int. 2026 Jun 15;39:16339. doi: 10.3389/ti.2026.16339 (PMC13310807; doi:10.3389/ti.2026.16339)

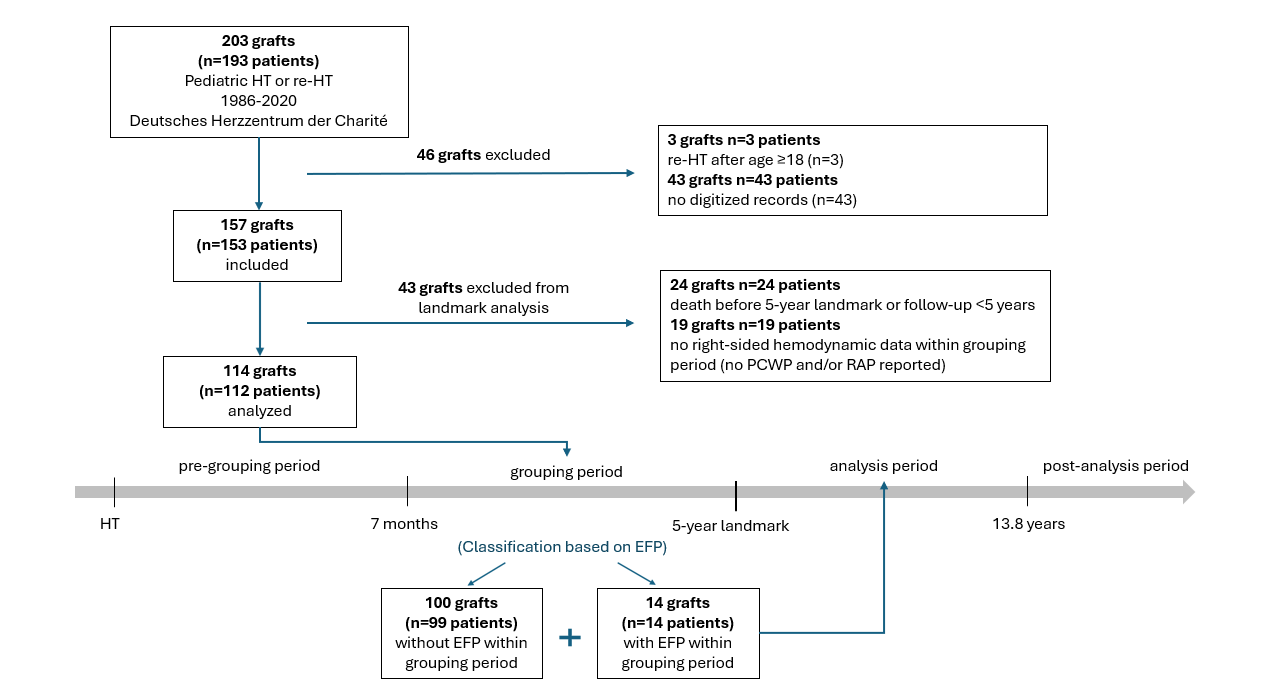

Supplement: Supplementary file 1 [file Image1.tiff]
