# Supplementary material for: Elevated filling pressures are associated with poor long-term graft survival after pediatric heart transplantation
Source: Transpl Int. 2026 Jun 15;39:16339. doi: 10.3389/ti.2026.16339 (PMC13310807; doi:10.3389/ti.2026.16339)
Supplement: Supplementary file 2 [file Supplementaryfile1.docx]

**Supplementary Tables**

**Table S1:** Demographic and clinical characteristics of included and excluded grafts

| ***Variables:*** | ***Total***  (n=157) | ***Analysis Set***  (n=114) | ***Excluded***  (n= 43) | ***p-value*** |
| --- | --- | --- | --- | --- |
| Recipients´ age at HT/re-HT, years | 8.3  (2.7 - 13.9) | 8.9  (2.7 - 14.3) | 6.2  (2.7 - 12.5) | 0.189 |
| Post-transplant follow up, years | 12.3  (7.2 - 17.7) | 13.1  (8.9 - 17.7) | 4.6  (3.6 - 17.5) | <0.001 |
| Donors´ age, years | 9  (3 - 25) | 9  (3 - 24) | 9.5  (3 – 26.8) | 0.920 |
| Donor/recipient age difference, years | 2  (-0.4 - 10.2) | 1.8  (-0.7 - 8.7) | 3.5  (-0.2 - 12.5) | 0.158 |
| Gender  Female | 73 (47%) | 51 (45%) | 22 (51%) | 0.589 |
| Number of re-HT* | 5 (3%) | 3 (3%) | 2 (5%) | 0.894 |
| *Eras of transplantation:*   - Early Era (1986 – 1999) - Late Era (2000 – 2020) | 47 (30%)  110 (70%) | 34 (30%)  80 (70%) | 13 (30%)  30 (70%) | 1.000 |
| *Cardiac diagnosis prior HT:*   - Cardiomyopathy - Congenital Heart Disease - Others - Re-HT | 123 (78%)  24 (15%)  5 (3%)  5 (3%) | 94 (83%)  14 (12%)  3 (3%)  3 (3%) | 29 (67%)  10 (23%)  2 (5%)  2 (5%) | 0.245 |
| Number of catheterizations per graft | 6  (4 - 9) | 7  (5 - 9) | 4  (3 - 6) | <0.001 |
| *LVEF echocardiographic evaluation***   - Grafts with preserved pLVEF ≥ 50% - Grafts with mildly reduced mrLVEF= 40-49% - Grafts with reduced rLVEF <40% | 116 (74%)  20 (13%)  21 (13%) | 81 (71%)  17 (15%)  16 (14%) | 35 (81%)  3 (7%)  5 (12%) | 0.345 |
| *Acute rejection episodes ≥ 1****   - From HT to the first catheterization with FP - Thereafter to the last follow-up or Death/re-HT | 44 (28%)  13 (8%)  31 (20%) | 41 (36%)  11 (10%)  30(26%) | 3 (7%)  2 (5%)  1 (2%) | <0.001 |
| Post-Transplant Lymphoproliferative Disorder | 15 (10%) | 11 (10%) | 4 (9%) | 1.000 |
| *Cardiac deaths:*   - CAV and/or acute rejection - Heart failure   *Noncardiac deaths*:   - PTLD - Infection/Sepsis   *Unknown* | 18 (12%)  11 (7%)  2 (1%)  6 (4%)  19 (12%) | 13 (11%)  8 (7%)  1 (1%)  2 (2%)  17 (15%) | 5 (12%)  3 (7%)  1 (2%)  4 (9%)  2 (5%) | 0.092 |

CAV = coronary allograft vasculopathy, EFP = Elevated filling pressures, FP = Filling pressures HT = Heart transplantation, LVEF = left ventricular ejection fraction, PTLD = Post transplant lymphoproliferative disorder, re-HT = Re-transplantation

*With event counts lower than 5, p-value violates assumption of chi-square test.

**For LVEF, values indicate the number of grafts categorized according to their lowest recorded EF during follow-up: pLVEF: preserved, always ≥ 50%, mrLVEF: mildly reduced, at least once <50% but never <40%, rLVEF: reduced, at least once <40% over the whole study period

***For acute rejection, values indicate the number of grafts that experienced ≥ 1 biopsy-proven episodes (≥2R/3A ACR and/or AMR).

**Table S2:** Cardiovascular adverse events within the grouping period in included and excluded grafts

| ***Variables:*** | ***Total***  (n=157) | ***Excluded***  (n= 43) | ***Analysis set***  (n=114) | ***p-value*** |
| --- | --- | --- | --- | --- |
| ***Within grouping period (7 months - 5 years post-transplant) **** | | | | |
| *Cardiovascular AE Composite Endpoint* ≥1:   - Moderate - severe epicardial CAV_2-3_ - Cardiac arrhythmia - Non-rejection heart failure hospitalization - MI and/or coronary revascularization | 15 (10%)  6 (4%)  5 (3%)  3 (2%)  7 (5%) | 3 (7%)  1 (2%)  -  2 (5%)  1 (2%) | 12 (11%)  5 (4%)  5 (4%)  1 (1%)  6 (5%) | 0.711  0.894  0.376  0.375  0.718 |

AE = Adverse events, CAV = coronary allograft vasculopathy, HT = Heart transplantation, MI = myocardial infarction
*Values represent the number of grafts (n) with at least one event, with percentages (%) referring to the respective group. Each graft is counted once per event category. Grafts may have experienced more than one event type, and multiple events within the same graft were possible.

**Table S3:** Demographic and clinical characteristics of EFP and never EFP grafts

| ***Variables:*** | ***Total***  (n=74) | ***Never EFP group***  (n=60) | ***EFP group***  (n=14) | ***p-value*** |
| --- | --- | --- | --- | --- |
| Recipients´ age at HT/re-HT, years | 8.5  (2.6 - 14.1) | 7.6  (2.5 - 13.6) | 12.2  (2.7 - 14.5) | 0.659 |
| Post-transplant follow up, years | 11.3  (8.0 - 16.2) | 12.4  (8.2 - 17.4) | 9.7  (6.7 - 11.1) | 0.016 |
| Donors´ age, years | 8.5  (3 - 25) | 8  (4 - 23.2) | 12  (3- 31.2) | 0.885 |
| Donor/recipient age difference, years | 1.8  (-0.4 - 10.6) | 1.8  (-0.4 - 8.6) | 1.6  (-0.1 - 15.5) | 0.751 |
| *Gender*  Female | 30 (41%) | 26 (43%) | 4 (29%) | 0.477 |
| Number of re-HT | 1 (1%) | - | 1 (7%) | 0.424 |
| *Eras of transplantation:*   - Early Era (1986 – 1999) - Late Era (2000 – 2020) | 17 (23%)  57 (77%) | 14 (23%)  46 (78%) | 3 (21%)  11 (79%) | 1.000 |
| *Cardiac diagnosis prior HT:*   - Cardiomyopathy - Congenital Heart Disease - Others - Re-HT | 62 (84%)  10 (14%)  1 (1%)  1 (1%) | 50 (83%)  10 (17%)  -  - | 12 (86%)  -  1 (7%)  1 (7%) | 0.012 |
| Time from HT to first catheterization with available FP, years | 1.3  (1.0 - 2.5) | 1.4  (1.0 - 2.9) | 1.1  (1.0 - 1.9) | 0.291 |
| Number of catheterizations per graft | 6  (4 - 9) | 6  (4 - 7) | 9  (7 - 15) | 0.001 |
| *LVEF echocardiographic evaluation**   - Grafts with preserved pLVEF ≥ 50% - Grafts with mildly reduced mrLVEF= 40-49% - Grafts with reduced rLVEF <40% | 57 (77%)  10 (14%)  7 (10%) | 48 (80%)  9 (15%)  3 (5%) | 9 (64%)  1 (7%)  4 (29%) | 0.023 |
| *Acute rejection episodes ≥ 1***   - From HT to the first catheterization with FP - Thereafter to the last follow-up or death/re-HT | 20 (27%)  5 (7%)  15 (20%) | 15 (25%)  4 (7%)  11 (18%) | 5 (36%)  1 (7%)  4 (29%) | 0.632 |
| Post-Transplant Lymphoproliferative Disorder | 7 (10%) | 7 (12%) | - | 0.403 |
| *Cardiac deaths:*   - CAV and/or acute rejection - Heart failure   *Noncardiac deaths*:   - PTLD - Infection/Sepsis   *Unknown* | 4 (5%)  2 (3%)  1 (1%)  1 (1%)  15 (20%) | 3 (5%)  1 (2%)  1 (2%)  -  10 (17%) | 1 (7%)  1 (7%)  -  1 (7%)  5 (36%) | 0.593 |

CAV = coronary allograft vasculopathy, EFP = Elevated filling pressures, FP = Filling pressures HT = Heart transplantation, LVEF = left ventricular ejection fraction, PTLD = Post-transplant lymphoproliferative disorder, re-HT = Re-transplantation

* For LVEF, values indicate the number of grafts categorized according to their lowest recorded EF during follow-up: pLVEF: preserved, always ≥ 50%, mrLVEF: mildly reduced, at least once <50% but never <40%, rLVEF: reduced, at least once <40% over the whole study period

**For acute rejection, values indicate the number of grafts that experienced ≥ 1 biopsy-proven episodes (≥2R/3A ACR and/or AMR).

**Table S4:** Cardiovascular adverse events within the grouping period in EFP and never EFP grafts

| ***Variables:*** | ***Total***  (n=74) | ***Never EFP group***  (n=60) | ***EFP group***  (n=14) | ***p-value*** |
| --- | --- | --- | --- | --- |
| ***Within grouping period (7 months - 5 years post-transplant) **** | | | | |
| *Cardiovascular AE Composite Endpoint* ≥1:   - Moderate - severe epicardial CAV_2-3_ - Cardiac arrhythmia - Non-rejection heart failure hospitalization - MI and/or coronary revascularization | 9 (12%)  4 (5%)  4 (5%)  1 (1%)  4 (5%) | 4 (7%)  -  4 (7%)  -  - | 5 (36%)  4 (29%)  -  1 (7%)  4 (29%) | 0.011  <0.001  0.736  0.424  <0.001 |

AE = Adverse events, CAV = coronary allograft vasculopathy, HT = Heart transplantation, MI = Myocardial infarction
*Values represent the number of grafts (n) with at least one event, with percentages (%) referring to the respective group. Each graft is counted once per event category. Grafts may have experienced more than one event type, and multiple events within the same graft were possible.

**Table S5:** Sensitivity Analysis: Adverse Event Rate Ratios based on negative binomial distribution

| **Variables** | **Rate Ratio** | **CI** |
| --- | --- | --- |
| *Composite Cardiovascular AE*   - Significant moderate - severe epicardial CAV_2-3_ - Cardiac arrhythmia - Non-rejection heart failure hospitalization - MI and/or coronary revascularization | 1.2  0.8  2.1  0.6  0.4 | [-0.3 - 3.4]  [-0.9 - 3.3]  [-0.6 - 7.6]  [-2.4 - 7.8]  [-1.7 - 3.1] |

AE = Adverse events, CAV = coronary, MI = Myocardial infarction

**Supplementary Figure Legends:**

**Supplementary Figure 1:** **Flowchart of the selection process for patients and their grafts included in the final analysis**.

Grafts were screened from all pediatric heart transplant recipients between 1986 and 2020. Of 203 pediatric heart transplant or re-transplant grafts, 114 grafts in 112 patients were included in the final analysis. Elevated filling pressure status was determined during the grouping period, defined as 7 months to 5 years after transplantation, based on available PCWP and/or RAP measurements. Grafts not reaching the 5-year landmark or lacking right-sided hemodynamic data during the grouping period were excluded. Survival analyses were performed from the 5-year landmark to 13.8 years after transplantation. The timeline illustrates the pre-grouping, grouping, analysis, and post-analysis periods.

**Capsule Sentence Summary:**

Elevated filling pressures 7 months to 5 years after pediatric heart transplantation were associated with worse graft survival and an overall sixfold higher risk of graft loss, with exploratory evidence of higher rates of cardiovascular complications.
